# Bibliography

**Published:** 1893-02

**Authors:** 


### EDITORIAL.

### Bibliography.

ELEMENTS OF CHEMISTRY AND DENTAL MATERIA MEDICA, by J. S. Cassidy, D.D.S., M.D., Professor of Chemistry and Materia Medica in the Ohio College of Dental Surgery, containing 364 pages.

This work just issued from the press of Robert Clarke & Co., of Cincinnati, is one that will serve a valuable purpose as a text-book in dental colleges. In this, Prof. Cassidy has accomplished a good work not only for the colleges and students, but for the profession as well. Having been a successfnl teacher in the Ohio College of Dental Surgery for well nigh a quarter of a century, he was well prepared for the preparation of such a work, knowing full well the needs of the student from practical experience.

The following statements in the preface will indicate clearly the scope of the work: "The labor involved in the preparation of the following pages was assumed at the solicitation of members of my classes; and also for the purpose of testing the comparative value to dental students of the two approved didactic methods of teaching chemistry and materia medica, viz: by lectures and quizzes and by recitation from approved text-books in relation with suggestive experiments. The division of the volume into three parts was made with a view to the convenience of the three graded classes, which now obtain, as required by the Association of Dental Faculties. The space given to the consideration of Chemical Philosophy and Compound Radicals, as well as that occupied by the few simple rules to be observed in prescription writing, and the approximate relations between the Metric and Apothecary's systems of weights and measures will not I trust be regarded as superfluous; for, dental students, as well as those of medicine, are often sadly deficient in a clear knowledge of those important accomplishments."

By a casual examination of the work we are led to the conclusion that it is a most excellent one for the purpose for which it was written. The Doctor does not lay claim to any special originality in the work; he gives the names of a number of authors to whose writings he is more or less indebted. He has adopted an innovation in the orthography of a number of words, which we regard as a step in the right direction; for instance, dropping the final "e" in quite a number of words; for example: Chlorin, Iodin, Glycerin, Cocain, Oxid, etc.

Each part of the work is well adapted for the class of students for which it is intended. Though necessarily brief, it will not only, if well studied, give the student the elementary principles, but will incite in him a desire to know more and pursue further these very important branches.

The work is one we can cordially recommend to the student and to the practitioner as well.

